# Associations Between Shift Work, Sociodemographic and Lifestyle Characteristics, Body Measurements, and MASLD

**DOI:** 10.3390/life15060961

**Published:** 2025-06-16

**Authors:** Javier Tosoratto, Pedro Juan Tárraga López, Ángel Arturo López-González, Carla Busquets-Cortes, Joan Obrador de Hevia, José Ignacio Ramirez-Manent

**Affiliations:** 1 Investigation Group ADEMA SALUD, University Institute for Research in Health Sciences (IUNICS), 07010 Palma, Balearic Islands, Spain; javiertosoratto@gmail.com (J.T.); c.busquets@eua.edu.es (C.B.-C.); j.obrador@eua.edu.es (J.O.d.H.); jignacioramirez@telefonica.net (J.I.R.-M.); 2 Faculty of Medicine, University of Castilla La Mancha (UCLM), 02008 Albacete, Spain; pjtarraga@sescam.jccm.es; 3 Health Service of Castilla La Mancha (SESCAM), 02008 Albacete, Spain; 4 Faculty of Dentistry, University School ADEMA, 07010 Palma, Balearic Islands, Spain; 5 Institut d’Investigació Sanitària de les Illes Balears (IDISBA), Balearic Islands Health Research Institute Foundation, 07010 Palma, Balearic Islands, Spain; 6 Balearic Islands Health Service, 07010 Palma, Balearic Islands, Spain; 7 Faculty of Medicine, University of the Balearic Islands, 07010 Palma, Balearic Islands, Spain

**Keywords:** shift work, MASLD, metabolic health, liver indices, sociodemographic factors, lifestyle habits

## Abstract

**Background:** Metabolic dysfunction-associated steatotic liver disease (MASLD) is the most prevalent chronic liver disorder worldwide and is closely linked to the components of metabolic syndrome. Shift work, through its disruption of circadian rhythms and the promotion of adverse behavioral patterns, has been proposed as a potential contributor to metabolic dysfunction and liver disease, yet evidence on its association with MASLD remains limited in large, heterogeneous occupational populations. **Objectives:** To investigate the association between shift work and MASLD risk using multiple validated non-invasive indices in a large sample of Spanish workers, and to explore the influence of sociodemographic characteristics, lifestyle behaviors, and sex on these associations. **Methods:** This cross-sectional study analyzed data from 53,053 employed adults across diverse sectors in Spain, including 31,753 men and 21,300 women. The participants underwent standardized occupational health assessments between 2019 and 2020. The MASLD risk was evaluated using seven indices: fatty liver index (FLI), hepatic steatosis index (HSI), ZJU index, fatty liver disease (FLD) index, Framingham steatosis index (FSI), lipid accumulation product (LAP), and BARD score. Sociodemographic, anthropometric, clinical, biochemical, and lifestyle data were collected. Multinomial logistic regression models were used to assess independent associations between shift work and high-risk MASLD scores. **Results:** Shift workers exhibited significantly higher mean values and prevalence of elevated MASLD scores across all indices compared to non-shift workers, in both sexes. In men, the prevalence of high BARD scores increased from 43.5% (non-shift) to 71.5% (shift), while in women it rose from 49.9% to 85.7%. Multivariate analysis confirmed that shift work was independently associated with an increased MASLD risk, particularly for HSI (OR: 7.83; 95% CI: 7.40–8.26) and ZJU (OR: 5.91; 95% CI: 5.60–6.22). Male sex, older age, smoking, and blue-collar status were also consistently associated with elevated risk scores. **Conclusions:** Shift work is significantly associated with an increased MASLD risk, independent of sociodemographic and lifestyle factors. Women and blue-collar workers may be especially vulnerable to the hepatic consequences of circadian disruption. These findings support the inclusion of liver health screening in occupational health programs and highlight the need for targeted interventions to reduce the MASLD risk among shift-working populations. Cross-sectional design limits causality; longitudinal studies are needed to confirm temporal relationships.

## 1. Introduction

Metabolic dysfunction-associated steatotic liver disease (MASLD), formerly known as non-alcoholic fatty liver disease (NAFLD), is a highly prevalent chronic liver condition that affects over 30% of the global population, posing a significant public health burden [1] and imposing considerable economic costs on healthcare systems [2]. It is characterized by excessive hepatic fat accumulation (hepatic steatosis) in the absence of significant alcohol intake or other secondary causes of liver disease [3]. MASLD arises from complex interactions among genetic, environmental, and cardiometabolic factors, with insulin resistance and adipose tissue dysfunction playing central pathophysiological roles. The condition encompasses a broad clinical spectrum, ranging from simple steatosis to metabolic dysfunction-associated steatohepatitis (MASH), formerly referred to as non-alcoholic steatohepatitis (NASH) [4], hepatic fibrosis [5], and, in more advanced stages, cirrhosis [6] and hepatocellular carcinoma [7]. Due to its strong association with metabolic syndrome [8] and other non-communicable chronic diseases, MASLD represents a research priority for the development of effective preventive and therapeutic strategies. Despite the recent advances in understanding its pathophysiology, the disease progression and treatment response remain heterogeneous. In this context, the Liver Disease Nomenclature Development Initiative proposed a more accurate and less stigmatizing terminology to better reflect the underlying causes of the disease, improve the diagnostic clarity, raise awareness, and facilitate more appropriate allocation of resources for research and clinical care [9].

The escalating prevalence of MASLD parallels the global rise in obesity and sedentary behaviors, but it is also increasingly evident that social determinants of health and work-related factors play a crucial role in shaping metabolic outcomes. In particular, shift work—a form of employment scheduling that diverges from the traditional diurnal work pattern—has been associated with circadian rhythm disruption, metabolic dysregulation, and the increased risk of cardiometabolic diseases [10,11]. Shift work includes night shifts, rotating schedules, and irregular hours, and it is prevalent in essential sectors such as healthcare, manufacturing, transportation, and security. The chronobiological alterations induced by shift work, including sleep deprivation, hormone imbalance, and altered eating patterns, may exacerbate metabolic dysfunction and inflammation, thus potentiating the development of MASLD [12,13].

Several studies have established associations between shift work and the components of metabolic syndrome, including obesity, hypertension, impaired glucose metabolism, and dyslipidemia [14,15,16]. However, relatively few large-scale epidemiological investigations have explored the potential link between shift work and MASLD, particularly using validated non-invasive indices of hepatic steatosis. Moreover, previous research has often focused on specific occupational groups or lacked detailed sociodemographic and lifestyle data, limiting the generalizability of findings and impeding the development of comprehensive preventive strategies.

Importantly, the influence of shift work on the MASLD risk may not be uniform across population subgroups. Sociodemographic characteristics such as sex, age, and occupational category, as well as health behaviors including smoking, alcohol consumption, diet, and physical activity, may modulate the relationship between work schedule and liver health. Blue-collar workers, who are more likely to be engaged in manual labor and irregular work hours, may face compounded risks due to higher rates of smoking, poor diet, and limited access to healthcare services [17,18]. Women, on the other hand, may exhibit differential susceptibility to metabolic disturbances depending on their hormonal status, caregiving responsibilities, and social stressors. As such, disaggregated analyses by sex and employment sector are essential to capture the full complexity of MASLD risk profiles in the context of shift work.

Given the subclinical nature of MASLD in its early stages, the use of surrogate biomarkers and composite indices is indispensable for population-level screening. A number of non-invasive scoring systems have been developed to estimate the presence of hepatic steatosis or fibrosis using routine clinical and biochemical parameters. These include the fatty liver index (FLI) [19], hepatic steatosis index (HSI) [20], Zhejian University (ZJU) index [21], fatty liver disease (FLD) index [22], lipid accumulation product (LAP) [23], and the BARD score for liver fibrosis [24]. Such tools provide valuable epidemiological insights and allow for the identification of at-risk individuals in occupational health settings without the need for imaging or liver biopsy.

Despite the availability of these validated indices, few studies have systematically applied multiple MASLD risk scales to large, heterogeneous working populations. This approach offers the advantage of cross-validation and may reveal distinct patterns depending on the components included in each index. For example, indices incorporating waist circumference and triglycerides may be more sensitive to dietary and physical activity changes, while those emphasizing AST/ALT ratios may better reflect liver-specific injury. Moreover, the comparison of these indices across work schedules and sociodemographic strata can shed light on the potential mechanisms and intervention targets.

In the present study, we sought to evaluate the association between shift work and the risk of MASLD in a large, diverse cohort of Spanish workers. Utilizing data from over 53,000 employed adults undergoing routine occupational health examinations across multiple regions and sectors, we examined six established MASLD indices to assess the potential impact of shift work on liver health. Our objectives were fourfold: (1) to compare sociodemographic characteristics, anthropometric measurements, clinical and biochemical markers, and lifestyle factors between shift and non-shift workers; (2) to analyze the mean values and prevalence of high-risk scores for each MASLD index according to shift status and sex; (3) to evaluate the independent contribution of shift work to an elevated MASLD risk through multivariable logistic regression models adjusting for key confounders; and (4) to explore the modifying role of age, sex, occupational class, and lifestyle behaviors on these associations.

To our knowledge, this is one of the largest occupational health studies to date examining the relationship between shift work and MASLD using a comprehensive panel of non-invasive indices. The integration of detailed demographic, anthropometric, biochemical, and lifestyle data allows for a nuanced understanding of the interplay between work patterns and liver health. Furthermore, the inclusion of both male and female workers across white- and blue-collar occupations enables the identification of vulnerable subgroups and informs tailored intervention strategies. The findings from this study hold relevance for occupational medicine practitioners, public health policymakers, and employers aiming to mitigate the metabolic risk in shift-working populations.

Each of the MASLD indices used in this study captures different aspects of hepatic pathology. For instance, the FLI and LAP primarily emphasize anthropometric and lipid parameters, making them sensitive to adiposity and dyslipidemia. The HSI and ZJU indices include liver enzyme ratios (e.g., AST/ALT), which better reflect hepatocellular injury. The FLD and FSI incorporate broader metabolic indicators, while the BARD score combines the AST/ALT ratio, BMI, and diabetes status to estimate the fibrosis risk. These differences may account for the varying strength of the associations observed among the indices, highlighting the value of a multimodal assessment approach.

In summary, this study addresses a critical gap in the literature by providing robust evidence on the association between shift work and MASLD in a representative working population. In the context of increasing MASLD prevalence and rising demands for non-standard work schedules, understanding the occupational determinants of liver disease is imperative for effective prevention and health promotion. The use of validated indices for early detection, coupled with targeted workplace interventions, may offer a pathway to reduce the burden of MASLD and improve the long-term outcomes among at-risk workers.

## 2. Methods

### 2.1. Participants

This study was designed as an observational, descriptive, and cross-sectional analysis involving a total of 53,053 workers from a broad range of employment sectors across different regions of Spain. The sample included 31,753 male participants (of whom 17,527 engaged in shift work) and 21,300 female participants (11,281 of whom also worked shifts). All the participants had undergone annual occupational health evaluations as part of their employment within the collaborating companies. The data collection period extended from January 2019 through June 2020.

The inclusion criteria were as follows:

- Age between 18 and 69 years.
- Active employment under contract with one of the participating organizations.
- Provision of informed consent for study participation.
- Authorization for the use of personal health data for epidemiological research.

**Figure 1:**
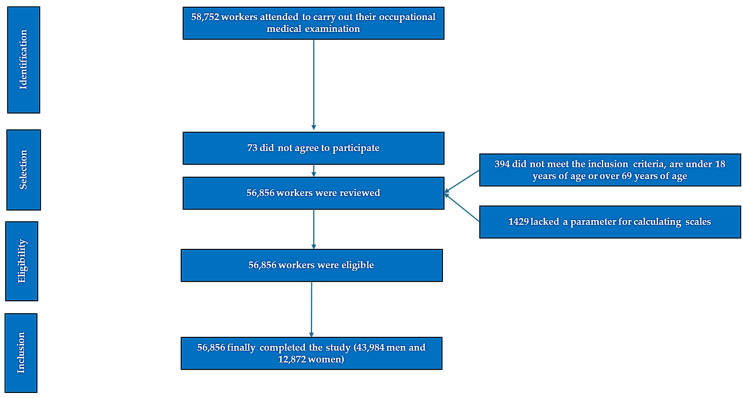
Flowchart of the participants.

A flowchart summarizing the application of the inclusion criteria and the selection process is presented in Figure 1.

### 2.2. Variable Assessment

Data were obtained by trained staff from the occupational health departments of the participating companies. Information was gathered primarily through clinical interviews and anamnesis. The dataset comprised variables related to sociodemographic characteristics (age, sex, educational attainment, and socioeconomic classification) and lifestyle behaviors (smoking, alcohol intake, adherence to the Mediterranean diet, and physical activity levels).

The clinical and anthropometric data collected included body weight, height, waist circumference, and systolic and diastolic blood pressure. Biochemical parameters involved fasting glucose and lipid profile markers.

Standard protocols were followed to ensure consistency in the measurement techniques and to reduce inter-observer variability.

### 2.3. Anthropometric Assessment

The participants’ height and weight were measured with minimal clothing and in a relaxed, upright posture, using a SECA-brand stadiometer and scale, in line with the guidelines from the International Society for the Advancement of Kinanthropometry (ISAK) [25]. Waist circumference was measured midway between the lowest rib and the iliac crest, and hip circumference was recorded at the widest part of the gluteal region, using a SECA flexible tape.

### 2.4. Clinical Measurements

Blood pressure was assessed using an OMROM-M3 device (OMRON, Osaka, Japan) after the participant had rested in a seated position for at least ten minutes. Measurements were taken three times at one-minute intervals, and the average value was used for analysis. Hypertension was defined as systolic pressure ≥140 mmHg, diastolic pressure ≥90 mmHg, or current antihypertensive treatment.

### 2.5. Laboratory Analysis

Fasting venous blood samples were collected after a minimum of 12 h without food intake. Samples were stored at 4 °C and analyzed within 48–72 h in certified laboratories following standardized procedures. Glucose and lipid levels (total cholesterol, HDL, LDL, and triglycerides) were measured using enzymatic methods, except HDL, which was assessed using precipitation techniques. LDL cholesterol was calculated via the Friedewald formula unless triglyceride levels exceeded 400 mg/dL, in which case direct measurement was employed. Dyslipidemia was defined based on laboratory reference values or the use of lipid-lowering medication.

### 2.6. Non Alcoholic Fatty Liver Disease Scales (Table 1)

In our study, we employed a variety of risk assessment scales specifically designed to evaluate the likelihood of metabolic dysfunction–associated steatotic liver disease (MASLD). These tools, chosen for their clinical relevance and validation, are comprehensively described in Table 1.

Sociodemographic and Lifestyle Variables

- Sex was recorded as male or female.
- Age was determined from the date of birth to the examination date.
- Educational level was categorized as primary, secondary, or tertiary (university) education.
- Social class was assigned based on the 2011 Spanish National Classification of Occupations (CNO-11) [26] following the Spanish Society of Epidemiology framework:
- Class I: University professionals, executives, elite athletes, and artists.
- Class II: Technicians and skilled self-employed workers.
- Class III: Manual laborers and less qualified workers.

Smoking status was defined as active use of tobacco within the past 30 days or failure to maintain abstinence for at least one year.

Dietary habits were assessed using a 14-item questionnaire to evaluate adherence to the Mediterranean diet. Each affirmative response scored one point; a total score ≥9 was classified as high adherence [27].

Physical activity was assessed via the International Physical Activity Questionnaire (IPAQ), which evaluates activity levels over the previous seven days [28].

Alcohol intake was expressed in Standard Drinking Units (SDUs), equivalent to 10 g of pure alcohol in Spain. Typical conversions included: 100 mL of wine or champagne, 200 mL of beer, or 25 mL of spirits. Consumption was considered high-risk if it exceeded 35 SDUs per week in men and 20 in women [29].

### 2.7. Statistical Analysis

Descriptive statistics were computed for the quantitative variables using means and standard deviations. Group comparisons were made using the Student’s *t*-test. For categorical variables, Chi-squared (χ^2^) tests were applied to estimate the prevalence. Multinomial logistic regression models were used to calculate odds ratios (ORs) and 95% confidence intervals (CIs). All analyses were conducted using IBM SPSS Statistics version 28.0. A significance level of *p* < 0.05 was applied throughout.

## 3. Results

Table 2 reveals the significant differences in anthropometric, clinical, and biochemical variables between shift and non-shift workers. Among men, shift workers were slightly younger and shorter but presented higher values of systolic and diastolic blood pressure, total cholesterol, fasting glucose, AST, ALT, and GGT. In women, shift work was also associated with a more unfavorable metabolic profile, including lower HDL cholesterol and higher waist circumference, triglycerides, glucose, and hepatic enzyme levels. Age distribution and occupational classification showed notable disparities: shift workers were more frequently younger and predominantly engaged in blue-collar occupations. These findings support the hypothesis that shift work contributes to the clustering of cardiometabolic risk factors, particularly in socioeconomically disadvantaged groups.

The mean values across all the MASLD indices (FLI, HSI, ZJU, FLD, FSI, LAP, and BARD) were consistently higher among shift workers, in both sexes. Although some differences were modest in absolute terms, their consistency across all the scales strengthens the observed association. Women showed lower absolute values overall; however, the relative increase associated with shift work was more pronounced, particularly in the BARD and LAP scores. This may indicate that women are more metabolically sensitive to the physiological disruptions caused by shift work (Table S1).

This table confirms a significantly higher prevalence of elevated risk scores in all the indices among shift workers. For example, in men, the proportion of individuals with high ZJU scores increased from 41.2% to 53.2%, and for BARD, from 43.5% to 71.5%. In women, the increases were even more striking, with high ZJU scores rising from 27.3% to 50.1% and BARD from 49.9% to 85.7%. These results not only reinforce the relationship between shift work and hepatic risk but also suggest potential sex-based differences in vulnerability (Table 3).

Importantly, although the group means for several indices were near the diagnostic thresholds, the prevalence data presented in Table 4 clarify that a substantial proportion of individuals exceeded the established high-risk cut-offs for each NAFLD index. These thresholds are widely accepted as indicative of a clinically relevant likelihood of MASLD (metabolic dysfunction-associated steatotic liver disease). For instance, among male shift workers, over 70% had a BARD score ≥2 and more than 50% surpassed the high-risk cut-off for the ZJU index. In women, the increases were similarly marked, with 85.7% showing elevated BARD scores and 50.1% exceeding the ZJU threshold. These findings confirm that the observed differences are not limited to borderline elevations but reflect a true excess in the proportion of high-risk individuals meeting the clinical criteria for suspected MASLD.

The multivariate analysis confirmed that shift work is independently associated with an increased risk in all the MASLD indices, with particularly high odds ratios for HSI (OR = 7.83) and ZJU (OR = 5.91). Male sex, older age, smoking, blue-collar status, and shift work were consistently associated with a higher probability of elevated risk scores. These associations highlight a cumulative effect of biological, behavioral, and social vulnerability factors converging to increase the liver disease risk among shift workers (Table 4).

## 4. Discussion

This study, which included over 53,000 workers from various professional sectors across Spain, provides robust evidence linking shift work to an increased risk of metabolic dysfunction-associated steatotic liver disease (MASLD). Utilizing six validated non-invasive indices (FLI, HSI, ZJU index, FLD index, FSI, LAP, and BARD score), our analysis demonstrated consistently higher risk scores among shift workers compared to non-shift workers. Moreover, significant differences were observed in anthropometric, clinical, and biochemical parameters. In male workers, those engaged in shift work exhibited higher systolic and diastolic blood pressures, and elevated levels of fasting glucose, total cholesterol, and liver enzymes, while among females, shift work was associated with lower HDL cholesterol and higher waist circumference and triglyceride levels. These findings underscore the hypothesis that non-standard work schedules may contribute to adverse metabolic profiles, ultimately increasing the risk of MASLD [30,31].

Prior research has established the association between shift work and metabolic derangements, including insulin resistance, dyslipidemia, and obesity [32]. However, few studies have specifically examined the relationship between shift work and MASLD using multiple screening indices simultaneously. Our findings extend the previous literature by confirming that shift work is an independent risk factor for MASLD, after the adjustment for potential confounders such as age, sex, socioeconomic status, and lifestyle habits [33]. In contrast to earlier studies that often focused on specific occupational groups or single risk markers, the present investigation adopts a comprehensive approach by integrating a suite of non-invasive indices. This methodological refinement enables cross-validation and provides more granular insights into the potential hepatic impact of shift work [34]. Furthermore, our data reveal that the prevalence of high-risk scores across indices is markedly greater among shift workers, corroborating earlier reports that linked circadian disruption to liver steatosis and fibrosis [35].

The pathophysiological mechanisms underlying the association between shift work and metabolic dysfunction-associated steatotic liver disease (MASLD) appear to be multifactorial. One of the primary mechanisms involves the disruption of the circadian rhythm [36]. Circadian misalignment—resulting from factors such as night shifts, exposure to artificial light during nighttime, and irregular sleep and feeding schedules—profoundly affects hepatic homeostasis, altering key processes such as lipid metabolism, insulin signaling, and inflammatory responses [37].

The liver is a highly circadian organ, regulated both by the central clock located in the suprachiasmatic nucleus (SCN) and by autonomous peripheral clocks influenced by factors like the feeding–fasting cycle. Recent studies have shown that desynchronization between these clocks disrupts the rhythmic expression of the key genes involved in lipogenesis and β-oxidation, such as Srebp-1c, Fasn, Acc1, Pparα, and Cpt1a. This dysregulation leads to ectopic lipid accumulation in the liver, predisposing individuals to hepatic steatosis and mitochondrial dysfunction [38]. Moreover, circadian disruption impairs insulin signaling by dysregulating proteins such as Akt, IRS-1, and FoxO1, thereby reducing the hepatic insulin sensitivity and promoting inappropriate gluconeogenesis, even in postprandial states.

This contributes to the development of hepatic insulin resistance, a key feature of metabolic syndrome and type 2 diabetes. From an inflammatory standpoint, the liver also displays circadian rhythmicity in the expression of cytokines and immune mediators. Circadian disruption fosters a pro-inflammatory environment, characterized by the activation of the NF-κB pathway, increased production of IL-6, TNF-α, and other pro-inflammatory cytokines, and enhanced infiltration of M1-polarized hepatic macrophages. This inflammatory microenvironment not only exacerbates liver injury but also further impairs insulin signaling, creating a vicious cycle of inflammation, insulin resistance, and metabolic dysfunction that promotes hepatic fat accumulation [39,40].

Evidence from murine models has demonstrated that the hepatocyte-specific deletion of circadian clock genes such as Bmal1 or Clock leads to severe disturbances in lipid and insulin metabolism, even in the absence of dietary modifications. In humans, circadian misalignment has been associated with altered metabolic profiles, particularly among night shift workers, who show a higher prevalence of non-alcoholic fatty liver disease (NAFLD), dyslipidemia, and hepatic inflammation [41]. Additionally, sleep deprivation—commonly observed in shift workers—has been linked to increased sympathetic activity, elevated cortisol levels, and systemic inflammation, all of which may further exacerbate liver injury and steatosis [42].

Altered dietary patterns and irregular meal timings, frequently seen in shift work populations, further contribute to metabolic disturbances. Erratic eating habits can result in postprandial hyperglycemia and dysregulated lipid profiles, which are critical in the development of hepatic steatosis. Moreover, shift workers often report lower consumption of healthy foods such as fruits and vegetables, coupled with a higher intake of processed and high-calorie foods [43]. These lifestyle factors, in conjunction with physical inactivity, synergistically increase the risk of MASLD [44]. Finally, the stress associated with irregular work schedules might also play a role; chronic stress can lead to endocrine abnormalities and increased pro-inflammatory cytokine production, promoting hepatic injury and fibrogenesis [45].

These findings underscore the importance of circadian alignment for liver health, suggesting that interventions such as time-restricted feeding, controlled light exposure, and regular sleep patterns may represent effective therapeutic strategies to restore hepatic rhythmicity and prevent chronic metabolic diseases.

Our analysis reveals notable differences in how shift work impacts male versus female workers, as well as distinctions based on occupational categories. Although the absolute risk scores for MASLD tend to be lower among women, the relative change associated with shift work is more pronounced. This could be due to hormonal differences that modulate metabolic responses or the dual burden of caregiving and occupational demands that many women experience [46]. Furthermore, blue-collar workers, who are more frequently exposed to shift work schedules, show a particularly adverse profile with higher risk scores and an increased prevalence of metabolic derangements [47]. These findings suggest that both biological sex and the nature of occupational roles may modify the impact of circadian disruption on liver health, warranting targeted preventive strategies for these vulnerable subgroups [48].

### 4.1. Strengths and Limitations

A major strength of this study is the extensive sample size, which allowed for detailed stratification by sex, age, and occupation. The application of multiple validated non-invasive indices to assess MASLD risk provides robust evidence and enables the cross-comparison of results. In addition, the inclusion of a wide range of variables—including detailed anthropometric, clinical, and biochemical measurements—allows for a comprehensive analysis of the factors contributing to hepatic risk [49].

Additionally, we adopted the updated MASLD terminology throughout the manuscript, as it better reflects the metabolic etiology of the disease and aligns with international consensus.

Nevertheless, there are limitations that must be acknowledged. First, the cross-sectional design precludes any inference of causality; therefore, while associations between shift work and MASLD risk are evident, longitudinal studies are needed to determine the temporal nature of these relationships. Second, although the use of non-invasive indices is advantageous for large-scale screenings, these tools are surrogates and do not replace imaging or histological examination for definitive MASLD diagnosis. Third, potential confounding factors such as diet quality and genetic predispositions may not have been fully accounted for despite the comprehensive dataset [50]. Finally, the study population consisted of Spanish workers, which might limit the generalizability of the findings to other ethnic or geographical populations [51].

### 4.2. Public Health and Occupational Implications

The high prevalence of MASLD among shift workers has significant implications for public health and occupational safety. Given that MASLD is not merely a liver condition but a multisystem disease associated with cardiovascular and metabolic complications, early identification and intervention in high-risk populations are crucial. Routine occupational health assessments should incorporate liver risk screening, especially for workers engaged in shift work and those in blue-collar sectors who may lack adequate health resources [52]. Policies aimed at reducing circadian disruption—such as optimizing shift schedules, ensuring adequate rest periods, and promoting healthy eating practices—may mitigate the adverse metabolic effects associated with non-standard work schedules. Moreover, targeted health education and preventive interventions could substantially improve the long-term outcomes for these workers [53].

### 4.3. Future Directions

Future research should aim to address the limitations identified herein through longitudinal and interventional studies. Prospective cohort studies would help determine the causal relationship between shift work and the progression of MASLD, while interventional studies could evaluate the efficacy of workplace modifications in reducing hepatic and metabolic risk [54]. Additionally, further exploration into the molecular pathways linking circadian rhythm disruption to MASLD development is warranted. Advances in genomics and metabolomics could provide new biomarkers for early detection and personalized intervention strategies [55]. Investigating the role of genetic susceptibility in modulating the response to shift work might also elucidate why some individuals are more vulnerable than others, offering opportunities for tailored preventive measures [56].

Furthermore, there is a need to diversify study populations to assess the impact of shift work on MASLD across different ethnic and socio-economic groups. Such efforts would help generalize the findings and support the development of universal occupational health guidelines. Finally, integrating multidisciplinary approaches that combine clinical, occupational, and public health perspectives could lead to more effective strategies for mitigating the impact of shift work on liver health [57].

## 5. Conclusions

In summary, our study provides robust evidence that shift work is significantly associated with an increased risk of MASLD, as determined by multiple validated non-invasive indices. The association remains significant after adjusting for potential confounders, indicating that shift work independently contributes to hepatic risk. Notably, women and blue-collar workers appear particularly vulnerable, highlighting the need for tailored interventions in these groups. Given the cross-sectional design of the study, causal relationships cannot be established; therefore, although the associations between shift work and MASLD risk are evident, longitudinal studies are required to clarify the temporal direction of these associations. These findings have important implications for occupational health policies, suggesting that routine screening and preventive strategies should be integrated into workplace health programs. Future longitudinal and mechanistic studies are warranted to further elucidate the causal pathways and to evaluate the effectiveness of targeted interventions in reducing the burden of MASLD among shift workers.

## Figures and Tables

**Table 1:** MASLD risk scales.

Index	Formula/Components	High-Risk Threshold
Fatty Liver Index (FLI)	FLI = [(e^(0.953 × ln(triglycerides) + 0.139 × BMI + 0.718 × ln(γ-GTP) + 0.053 × waist circumference − 15.745))/(1 + e^(0.953 × ln(triglycerides) + 0.139 × BMI + 0.718 × ln(γ-GTP) + 0.053 × waist circumference − 15.745))] × 100	≥60
Hepatic Steatosis Index (HSI)	HSI = 8 × (ALT/AST) + BMI + 2 (if diabetic) + 2 (if woman)	≥36
Zhejliang University Index (ZJU)	ZJU Index = BMI (kg/m^2^) + fasting plasma glucose (mmol/L) + triglyceride (mmol/L) + 3 × [AST(IU/L)/ALT(IU/L)] + 2 (if woman)	≥38
Faty Liver Disease Index (FLD)	FLD index = BMI + TG + 3 × (ALT/AST ratio) + 2 × HG (yes = 1, no = 0)	≥37
Framingham Steatosis Index (FSI)	FSI = −7.981 + 0.011 × Age − 0.146 × Sex (female = 1, male = 0) + 0.173 × BMI + 0.007 × Triglycerides + 0.593 × Hypertension (yes = 1, no = 0) + 0.789 × Diabetes (yes = 1, no = 0) + 1.1 × (ALT/AST ratio > = 1.33, yes = 1, no = 0)	Continuos
Lipid Accumulation Product (LAP)	Men:(WC (cm) − 65) × TG (mmol/L)); Women:(WC (cm) − 58) × TG (mmol/L))	≥42.7
BARD Score	BMI ≥ 28 = 1 point, (AST/ALT) ratio ≥ 0.8 = 2 points, type 2 diabetes mellitus = 1 point.	2–4 points

**Table 2:** Characteristics of the population.

	No Shift Work	Shift Work		No Shift Work	Shift Work	
	Men *n* = 7444	Men *n* = 5238		Women *n* = 4422	Women *n* = 6787	
	Mean (SD)	Mean (SD)	*p*-Value	Mean (SD)	Mean (SD)	*p*-Value
Age (years)	44.6 (8.1)	43.8 (10.9)	<0.001	42.4 (7.4)	42.0 (10.0)	0.011
Height (cm)	176.9 (5.7)	175.1 (6.7)	<0.001	165.8 (5.1)	162.5 (6.3)	<0.001
Weight (kg)	84.9 (14.9)	85.2 (12.1)	0.328	66.2 (11.6)	66.7 (11.0)	0.025
Waist (cm)	88.7 (9.2)	88.2 (10.8)	0.004	73.5 (8.4)	74.1 (9.1)	<0.001
Systolic BP (mmHg)	129.4 (14.1)	130.3 (17.0)	0.001	117.9 (15.1)	119.1 (15.7)	<0.001
Diastolic BP (mmHg)	78.8 (9.8)	79.2 (11.8)	0.029	72.6 (9.9)	72.7 (10.6)	0.650
Total cholesterol (mg(dL)	195.0 (38.3)	198.3 (35.3)	<0.001	190.8 (34.9)	192.2 (36.2)	0.031
HDL-cholesterol (mg/dL)	51.7 (11.4)	48.5 (8.3)	<0.001	64.9 (16.1)	56.0 (7.7)	<0.001
LDL-cholesterol (mg/dL)	120.4 (37.6)	121.6 (32.6)	0.050	110.2 (33.2)	115.3 (34.1)	<0.001
Triglycerides (mmHg)	128.5 (81.7)	133.2 (78.4)	0.001	85.7 (41.0)	97.9 (58.0)	<0.001
Glucose (mg/dL)	91.1 (17.6)	96.4 (25.4)	<0.001	86.6 (11.7)	89.9 (13.8)	<0.001
AST (U/I)	24.1 (10.3)	26.4 (14.9)	<0.001	19.8 (9.7)	22.8 (12.0)	<0.001
ALT (U/I)	31.0 (19.1)	35.9 (19.2)	0.008	17.8 (6.6)	20.2 (9.2)	<0.001
GGT (U/I)	35.7 (33.9)	38.3 (36.5)	<0.001	21.4 (16.5)	23.0 (25.5)	<0.001
	**%**	**%**	***p*-Value**	**%**	**%**	***p*-Value**
18–29 years old	3.1	11.8	<0.001	2.7	12.0	<0.001
30–39 years old	23.9	23.3		32.8	28.0	
40–49 years old	43.8	30.5		46.9	34.5	
50–59 years old	26.7	27.6		16.4	23.7	
60–69 years old	2.5	6.8		1.2	1.8	
White collar	97.3	0	<0.001	99.7	0.0	<0.001
Blue collar	2.7	100		0.3	100.1	
Non-smokers	69.2	68.5	0.203	65.7	64.7	0.129
Smokers	30.8	31.5		34.3	35.3	

**Table 3:** Prevalence of high values of different MASLD risk scales according shift work or non-shift work by sex.

		Men			Women	
	Non Shift Work *n* = 7444	Shift Work *n* = 5238		Non Shift Work *n* = 4422	Shift Work *n* = 6787	
	%	%	*p*-Value	%	%	*p*-Value
Fatty liver index high	28.2	32.2	<0.001	5.4	6.2	<0.001
Hepatic steatosis index high	58.6	65.6	<0.001	45.2	54.5	<0.001
Zhejian University index high	41.2	53.2	<0.001	27.3	50.1	<0.001
Fatty liver disease index high	65.6	73.4	<0.001	52.3	60.1	<0.001
Lipid accumulation product high	43.7	46.4	<0.001	20.4	27.1	<0.001
BARD score high	43.5	71.5	<0.001	49.9	85.7	<0.001

**Table 4:** Multinomial logistic regression.

	FLI High	HSI High	ZJU High	FLD High	LAP High	BARD High
	OR (95% CI)	OR (95% CI)	OR (95% CI)	OR (95% CI)	OR (95% CI)	OR (95% CI)
Female	1	1	1	1	1	1
Male	6.56 (5.93–7.26)	1.45 (1.20–1.75)	1.47 (1.21–1.79)	2.08 (1.91–2.25)	2.51 (2.37–2.66)	1.48 (1.39–1.57)
18–29 years old	1	1	1	1	1	1
30–39 years old	1.13 (1.10–1.16)	1.10 (1.06–1.15)	1.12 (1.06–1.18)	1.15 (1.08–1.22)	1.17 (1.08–1.27)	1.20 (1.09–1.29)
40–49 years old	1.44 (1.19–1.74)	1.51 (1.39–1.63)	1.29 (1.20–1.39)	1.26 (1.17–1.35)	1.51 (1.39–1.63)	2.39 (2.21–2.57)
50–59 years old	2.88 (2.35–3.53)	1.79 (1.62–1.97)	1.81 (1.68–1.94)	1.60 (1.43–1.77)	2.30 (1.95–2.70)	2.66 (2.41–2.91)
60–69 years old	4.61 (3.45–6.16)	2.09 (1.89–2.29)	2.48 (2.23–2.73)	1.99 (1.80–2.18)	3.16 (2.61–3.83)	3.13 (2.88–3.38)
White collar	1	1	1	1	1	1
Blue collar	3.06 (2.30–4.06)	6.88 (6.50–7.16)	8.12 (7.60–8.64)	2.18 (2.05–2.31)	2.07 (1.88–2.26)	1.45 (1.30–1.6)
Non-shift work	1	1	1	1	1	1
Shift work	2.45 (1.84–3.26)	7.83 (7.40–8.26)	5.91 (5.60–6.22)	2.53 (2.31–2.75)	1.57 (1.39–1.75)	3.83 (3.60–4.06)
Non-smokers	1	1	1	1	1	1
Smokers	1.10 (1.04–1.17)	1.08 (1.05–1.12)	1.09 (1.05–1.13)	1.18 (1.09–1.27)	1.05 (1.01–1.12)	1.23 (1.16–1.30)

## Data Availability

All the study data are securely stored in a database that complies with the institutional and legal data protection standards at ADEMA-Escuela Universitaria. Oversight of data protection is managed by the institution’s Data Protection Officer, Dr. Ángel Arturo López González.

## Supplementary Materials

The following supporting information can be downloaded at: https://www.mdpi.com/article/10.3390/life15060961/s1, Table S1: Mean values of different MASLD risk scales according shift work or non shift work by sex.
