# Supplementary material for: Associations Between Shift Work, Sociodemographic and Lifestyle Characteristics, Body Measurements, and MASLD
Source: Life (Basel). 2025 Jun 16;15(6):961. doi: 10.3390/life15060961 (PMC12194742; doi:10.3390/life15060961)
Supplement: Supplementary file 1 [file life-15-00961-s001.zip › life-3633340-supplementary.pdf]

Table S1. Mean values of different MASLD risk scales according shift work or non shift work by sex.

	Men			Women		
	**Non Shift Work n = 7444**	**Shift Work n=5238**		**Non Shift Work n = 4422**	**Shift Work n = 6787**	
	**Mean (SD)**	**Mean (SD)**	***p*-Value**	**Mean (SD)**	**Mean (SD)**	***p*-Value**
**Fatty liver index**	43.5 (25.6)	45.0 (27.8)	0.008	15.1 (17.9)	17.3 (18.9)	<0.001
**Hepatic steatosis index**	37.6 (6.0)	38.7 (6.3)	0.004	35.4 (4.8)	37.1 (6.7)	<0.001
**Zhejian University index**	37.7 (4.5)	38.5 (5.5)	0.007	35.5 (4.2)	36.4 (4.4)	0.007
**Fatty liver disease index**	32.8 (4.3)	33.3 (5.0)	0.064	28.8 (4.0)	29.7 (4.3)	0.010
**Framingham steatosis index**	0.16 (0.21)	0.32 (0.19)	<0.001	0.08 (0.05)	0.14 (0.11)	<0.001
**Lipid accumulation product**	36.7 (33.6)	37.2 (33.5)	0.388	1.1 (1.1)	2.3 (0.8)	<0.001
**BARD score**	1.2 (1.1)	2.0 (1.1)	<0.001	15.8 (14.4)	19.0 (19.9)	<0.001
